# Investigating the Interface between Ceramic Particles and Polymer Matrix in Hybrid Electrolytes by Electrochemical Strain Microscopy

**DOI:** 10.3390/nano12040654

**Published:** 2022-02-15

**Authors:** Philipp M. Veelken, Maike Wirtz, Roland Schierholz, Hermann Tempel, Hans Kungl, Rüdiger-A. Eichel, Florian Hausen

**Affiliations:** 1 Institute of Energy and Climate Research, IEK-9, Forschungszentrum Jülich, 52425 Jülich, Germany; ph.veelken@fz-juelich.de (P.M.V.); maike.wirtz@rwth-aachen.de (M.W.); r.schierholz@fz-juelich.de (R.S.); h.tempel@fz-juelich.de (H.T.); h.kungl@fz-juelich.de (H.K.); r.eichel@fz-juelich.de (R.-A.E.); 2 Institute of Physical Chemistry, RWTH Aachen University, Landoltweg 2, 52074 Aachen, Germany; 3 Jülich-Aachen Research Alliance, Section JARA-Energy, 52425 Jülich, Germany

**Keywords:** Atomic Force Microscopy, Electrochemical Strain Microscopy, hybrid electrolyte, Energy Storage, lithium transport, lithium distribution, all-solid-state electrolytes

## Abstract

The interface between ceramic particles and a polymer matrix in a hybrid electrolyte is studied with high spatial resolution by means of Electrochemical Strain Microscopy (ESM), an Atomic Force Microscope (AFM)-based technique. The electrolyte consists of polyethylene oxide with lithium bis(trifluoromethanesulfonyl)imide (PEO_6_–LiTFSI) and Li_6.5_La_3_Zr_1.5_Ta_0.5_O_12_ (LLZO:Ta). The individual components are differentiated by their respective contact resonance, ESM amplitude and friction signals. The ESM signal shows increased amplitudes and higher contact resonance frequencies on the ceramic particles, while lower amplitudes and lower contact resonance frequencies are present on the bulk polymer phase. The amplitude distribution of the hybrid electrolyte shows a broader distribution in comparison to pure PEO_6_–LiTFSI. In the direct vicinity of the particles, an interfacial area with enhanced amplitude signals is found. These results are an important contribution to elucidate the influence of the ceramic–polymer interaction on the conductivity of hybrid electrolytes.

## 1. Introduction

Over the last few years, the interest in all-solid-state batteries (ASSBs) has increased due to their enhanced safety and theoretical capacity compared to conventional organic, liquid electrolyte batteries [1,2,3]. Polymers, ceramics and polymer/ceramic hybrid materials are under development for application in ASSBs. Polymer electrolytes allow for improved electrolyte–electrode interfaces compared to ceramic-based electrolytes due to their higher mechanical flexibility [4,5,6]. However, polymer electrolytes display comparably low ionic conductivities [5,7]. In contrast to polymer electrolytes, ceramic electrolytes demonstrate superior conductivities [8,9,10].

Ceramic electrolytes are brittle and inherently exhibit a high rigidity. Furthermore, their interface towards electrodes is hindered due to their rough surface structure. The current research focuses on overcoming these limitations by employing hybrid electrolytes based on a polymer matrix and added ceramic particles. Hybrid electrolytes exhibit important advantages of multiple solid-state electrolyte types, such as superior electrode–electrolyte contact and flexibility [11,12,13,14].

Keller et al. gave an excellent overview of recent developments and problems regarding different hybrid electrolyte types [15]. With the addition of ceramic particles into the polymer electrolyte, the goal is to increase the global conductivity of the polymer electrolyte while retaining the flexibility.

The literature points to different conductivity tendencies in polymer electrolytes with added ceramic electrolyte particles. There are reports showing an increase in the ionic conductivity with added ceramic particles [16,17,18]. However, there are also studies showing the complete opposite—a decrease in the ionic conductivity when the polymer electrolyte is filled with ceramic particles [19,20]. Hence, hybrid electrolytes require careful optimization of the lithium salt concentration in order to achieve high ionic conductivities [21].

The lithium ion conductivities of the individual ceramic and polymer components in hybrid electrolytes are only two of the factors that determine the overall conductivity of a hybrid electrolyte. A pronounced influence on the conductivity of the hybrids results from the modification of the material in the vicinity of ceramic particles and from the transition resistance between polymer and ceramic components in the hybrid. The presence of ceramic particles prevents local poly(ethylene oxide) (PEO) chain organization and leads to a high degree of disorder in the polymer neighboring the ceramic particles [22,23,24].

Moreover, in the interface region between the polymer and ceramic particles, Lewis acid–base interactions with the electrolyte ionic species form and promote lithium salt dissociation [24]. Dixit et al. found, by simulations, that, inside composite materials, an interfacial area forms between bulk polymer phases and single ceramic particles [25,26].

Furthermore, they modeled the interfacial conductivity by Effective Mean Field Theory and stated that the interfacial conductivity depends on the composition of the hybrid electrolyte, as, for 25 wt.% Li_7_La_3_Zr_2_O_12_ (LLZO) in PEO the interfacial conductivity was lower than in a hybrid film with 75 wt.% LLZO in PEO. The transition of the lithium ions across the interface between the polymer and the ceramic determines the possible pathways for the lithium transport within the hybrid electrolyte. The activation energies for the ion transfer across the polymer/ceramic (PEO/LLZO) interface were up to 96 kJ/mol (0.9 eV) [27], and interface resistances within the hybrid material were high.

Information from typically applied methods, such as electrochemical impedance spectroscopy (EIS) and cyclic voltammetry can be collected as an average from the entire sample, i.e., globally [21,28]. On the other hand, information with a high spatial resolution, i.e., on a local scale, are required to identify the transport path for the lithium ions and to improve the materials and cell designs. In particular, the interactions between different types of materials, such as polymers and ceramics in hybrid electrolytes have to be understood in detail.

Zheng et al. employed isotopically labeled ^6^Li NMR in a LLZO-PEO hybrid electrolyte to observe the lithium diffusion inside cycled symmetrical battery cells [29]. After cycling, they found that, at a high ceramic content, the lithium attempted to primarily move through the ceramic phase. ^6^Li NMR was also applied by Li et al., showing comparable results in Li_10_GeP_2_S_12_-PEO [30]. For a lower ceramic content (10 wt.%), the main conduction pathway was through the polymer phase, while, with increasing ceramic content (>50 wt.%), the conduction pathway was mostly inside the ceramic phase.

Recently, based on NMR experiments, Ranque et al. suggested that the ion transport between the polymer and ceramic phase is possible also for low (10%) Li_6.55_Ga_0.15_La_3_Zr_2_O_12_ content while being, however, comparably slow [31].

In this study, Electrochemical Strain Microscopy (ESM) was employed to investigate the local ionic conductivity in a hybrid electrolyte. Typically, ESM is used on electrode materials [32,33]. Generally, in Electrochemical Strain Microscopy (ESM) an alternating voltage with the same frequency as the Contact Resonance Frequency (CRF) between the conductive tip and sample is applied in contact mode. The electrical field at the tip forces mobile lithium ions inside the material towards or away from the tip [34,35]. The signal origin of ESM in electrolytes was discussed recently.

Schön et al. showed on Li_1.3_Al_0.3_Ti_1.7_(PO_4_)_3_ (LATP) that the dominant contribution to the resulting ESM signal is caused by electrostatic forces [36]. A link between the chemical composition and local tip-sample interaction was found. The aim of this work is to investigate the interfacial area between ceramic particles and a surrounding polymer matrix, as the ion transport through this interfacial area is still under debate. Therefore, applying ESM on a hybrid all-solid-state electrolyte offers insights into the local ionic mobility and transport between different electrolyte materials.

## 2. Experimental Section

The preparation method of LI_6.5_La_3_Zr_1.5_Ta_0.5_O_12_ (LLZO:Ta) is described in detail in [21]. It is important to note that OH groups are present on the ceramic particles. The synthesis of the hybrid electrolyte was performed in inert gas atmosphere inside a glovebox (MBraun, Stratham, NH, USA) to ensure the exclusion of water and oxygen (H_2_O < 0.1 ppm, O_2_ < 0.1 ppm). The hybrid electrolyte film was synthesized using the solution-casting method with anhydrous acetonitrile inside the glovebox. The ratio of ethylene oxide monomer groups to lithium ions (Li:EO = 1:x) was used to define the LiTFSI (Sigma-Aldrich, St. Louis, MO, USA, 99.95%) concentration.

In this case, a film with a ratio of Li:EO = 1:6 was synthesized. We dispersed 50 wt.% LLZO:Ta powder with respect to PEO (MW = 1,000,000 g mol^−1^, Alfa Aesar, Ward Hill, MA, USA) in acetonitrile and added to the polyethylene oxide with lithium bis(trifluoromethanesulfonyl)imide (PEO_6_–LiTFSI) solution. For the PEO_6_–LiTFSI with 50 wt.% LLZO:Ta, as investigated here, no increase of the ionic conductivity in comparison to the pure polymer was observed by EIS [21]. For the Atomic Force Microscope (AFM) and ESM measurements, a Bruker Dimension Icon (Bruker, Santa Barbara, CA, USA) operating inside a glovebox (H_2_O < 0.1 ppm, O_2_ < 0.1 ppm, Argon filled) was used.

In the ESM mode, we tracked the contact resonance frequency and amplitude with a phase-locked loop (HF2LI, Zurich Instruments, Zurich, Switzerland). For the measurements, a cantilever with a free resonance peak around 75 kHz and a conductive platinum iridium (Pt/Ir) coating (PPP-EFM, nominal spring constant 2.8 N m^−1^, Nano World AG, Neuchatel, Switzerland) was utilized. A baseline correction was performed with the respective contact resonance frequencies. For the amplitude distribution, all peaks were fitted as Gaussian according to Equation (1):

(1) $$y=y_{0}+\frac{Ae^{\frac{-4ln\left(2\right){\left(x-x_{c}\right)}^{2}}{w^{2}}}}{w\sqrt{\frac{\pi }{4ln(2)}}}\;$$

where $y_{0}$ is the baseline, A is the area under the peak, w is the width of the peak and $x_{c}$ is the center of the peak. The Focused Ion Beam (FIB) polishing process was conducted with a NanoLab 460FI (FEI, Waltham, MA, USA). The sample transfer between the FIB and AFM was realized in an inert gas shuttle. The Scanning Electron Microscopy (SEM) image was recorded on the same material but from a different batch. The sample was broken in liquid nitrogen and measured with a FEI Quanta FEG 650 with an accelerating voltage of 10 kV utilizing a Back Scattered Electron (BSE) detector.

## 3. Results and Discussion

Esm measurements require a specifically prepared surface of the PEO_6_–LiTFSI film containing 50 wt.% LLZO:Ta to avoid cross-talk between topographical features and the ESM amplitude signal. Therefore, the solution-cast hybrid electrolyte film was carefully polished using a focused ion beam (FIB). For ESM experiments, it is of the utmost importance to clearly distinguish between isolated ceramic particles and the surrounding polymer matrix to identify the interfacial region between both materials. Individual particles within the polymer matrix were identified based on their respective frictional response.

**Figure 1:**
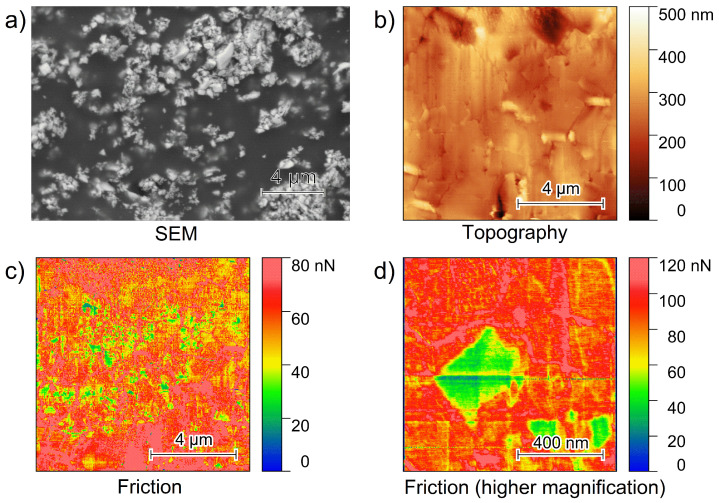
(**a**) SEM-BSE image of a PEO_6_–LiTFSI with 50 wt.% LLZO:Ta film, (**b**) 10 µm × 10 µm topography, (**c**) 10 µm × 10 µm friction image of a PEO_6_–LiTFSI with 50 wt.% LLZO:Ta film polished with a focused ion beam and (**d**) a higher magnification of a friction image showing a single ceramic particle within the polymer matrix.

For the interpretation of Esm signals, another important prerequisite is to always record the amplitude signal at the respective Contact Resonance Frequency (CRF) of the cantilever. However, variations in the mechanical properties strongly influence the resulting frequencies and ESM signals. Figure 1 shows a typical SEM-BSE image of a hybrid electrolyte film, while the AFM topography and friction images display the resulting smooth hybrid electrolyte film of the polished surface.

The SEM-BSE image depicted in Figure 1a exhibits multiple bright spots on an area of 20 µm × 14 µm, representing the high Z LLZO:Ta-particles. The dark area in the SEM image in Figure 1a reveals the PEO_6_–LiTFSI, as this material represents the matrix in which LLZO:Ta particles are embedded. Inside the polymer matrix, the LLZO:Ta particles tend to form agglomerates. In addition to the SEM image, a typical 10 µm × 10 µm AFM topography image is shown in Figure 1b. Vertical lines are observed from the ion milling process.

These effects are typical artifacts, known as curtaining, from the focused ion beam polishing process as this was executed from the top of the image to the bottom. The image Figure 1c illustrates a 10 µm × 10 µm friction image that was recorded simultaneously with Figure 1b. Within a homogeneous matrix exhibiting a friction value of 60–80 nN, several spots showing lower friction values are observed. A higher magnification of an isolated square-shaped particle exhibiting low friction values is shown in Figure 1d.

The friction images demonstrate the materials’ differences, similar to the SEM image of a different area. Lower friction values are associated with the ceramic particles, while higher friction values are assigned to the bulk polymer phase. Due to the softness of the PEO_6_–LiTFSI polymer and the stiffness of the ceramic, they show different tip–surface interactions. The friction image reveals some agglomerates of ceramic particles, i.e., lower friction values inside the bulk polymer phase as also detected by SEM with a particle size between 0.4 and 1 µm and a mean particle size of <1 µm [21].

**Figure 2:**
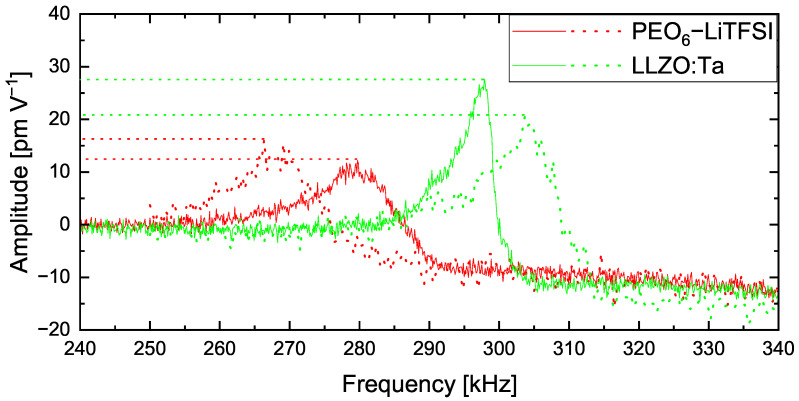
Contact resonance frequency curves on PEO_6_–LiTFSI (red) and LLZO:Ta (green).

Figure 2 shows CRF curves on individual spots on the polymer matrix PEO_6_–LiTFSI (red) and on LLZO:Ta ceramic particles (green). The CRF between the AFM tip and the LLZO:Ta particle is observed between 297 and 304 kHz. In comparison, the frequency of the identical tip in contact with the polymer matrix is found in the region of 266 to 279 kHz. Additionally, we observed that the polymer peaks are broader and exhibit lower amplitude values compared with the peaks obtained on the ceramic particle. The difference between the polymer peaks and ceramic peaks verifies the highly individual properties of both materials within the hybrid electrolyte film. As the polymer is a softer material than the ceramic particle, the damping of the cantilever causes the range to shift towards lower frequencies and become broader in comparison to the ceramic peaks [37].

This verifies that polymer matrix and ceramic particles can be differentiated by AFM-based techniques employing a sophisticated sample preparation method. This is especially relevant as the topography image does not show the materials’ differences.

### 3.1. Electrochemical Strain Signal Distribution on the Individual Polymer and Ceramic Electrolyte

**Figure 3:**
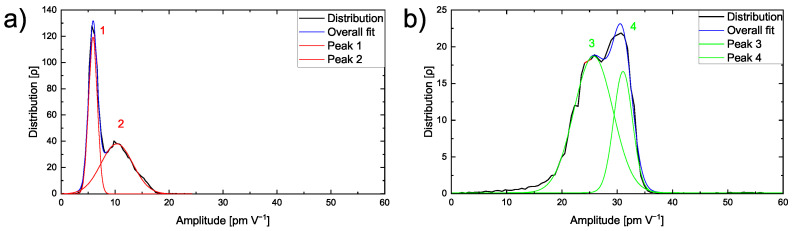
(**a**) Graph with the amplitude distribution of a 10 µm × 10 µm area on a pure PEO_6_–LiTFSI electrolyte film and (**b**) graph with the amplitude distribution of a 500 nm × 500 nm area on a LLZO:Ta pellet.

The amplitude distribution is studied on the single materials to differentiate the ESM amplitude signal on the pure PEO_6_–LiTFSI and a LLZO:Ta pellet. The lower amplitude of the polymer peak might be attributed to the lower lithium ion conductivity of the polymer compared to the ceramic electrolyte. A closer inspection of the amplitude signal on a 10 µm × 10 µm area of pure PEO_6_–LiTFSI reveals that it follows a bimodal distribution as depicted in the graph in Figure 3a. Two peaks exhibiting amplitude values of 5.9 pm V^−1^ and of 10.4 pm V^−1^ are found.

The first peak shows a Full-Width-Half-Maximum (FWHM) value of 1.9 pm V^−1^, while the second broader peak has a FWHM of 7.0 pm V^−1^. Higher amplitude values indicate a higher lithium mobility in this particular area. This observation illustrates heterogeneous lithium mobility in a polymer matrix in agreement with earlier observations and is discussed in literature [38,39]. The variation in lithium ion conductivity is typically caused by non-conducting crystalline regions and ion-conducting amorphous regions within the polymer. Therefore, the first peak is assigned to the crystalline phase, thus, resulting in a narrow FWHM. In comparison, the amorphous conducting phase is critically influenced by the inhomogeneity of the microstructure and, hence, shows a broader distribution in the second peak.

The amplitude distribution on a 500 nm × 500 nm area on a LLZO:Ta pellet is displayed in Figure 3b. The graph shows two distinguishable peaks, one at an amplitude value of 25.7 pm V^−1^ and the other at 31.1 pm V^−1^. The first peak is broader with a FWHM of 8.3 pm V^−1^, as the second peak is narrower with a FWHM of 4.0 pm V^−1^.

In comparison to the amplitude peaks on PEO_6_–LiTFSI, the LLZO:Ta peaks are found at higher amplitude values. This correlates with the different ionic conductivities of the PEO_6_–LiTFSI and LLZO:Ta electrolytes. The PEO_6_–LiTFSI shows lower amplitude values with a lower ionic conductivity, whereas the LLZO:Ta shows higher amplitude values with a higher ionic conductivity.

The two distinct peaks in the distribution of the LLZO:Ta ceramic particles might originate from microstructural or morphological effects influencing the local ionic conductivity within the material. To further understand the ESM amplitude signal response of the hybrid electrolyte film, a similar approach was conducted on the polished sample within an area exhibiting particles as well as the polymer matrix.

### 3.2. Electrochemical Strain Signal Distribution Inside the Hybrid Electrolyte

**Figure 4:**
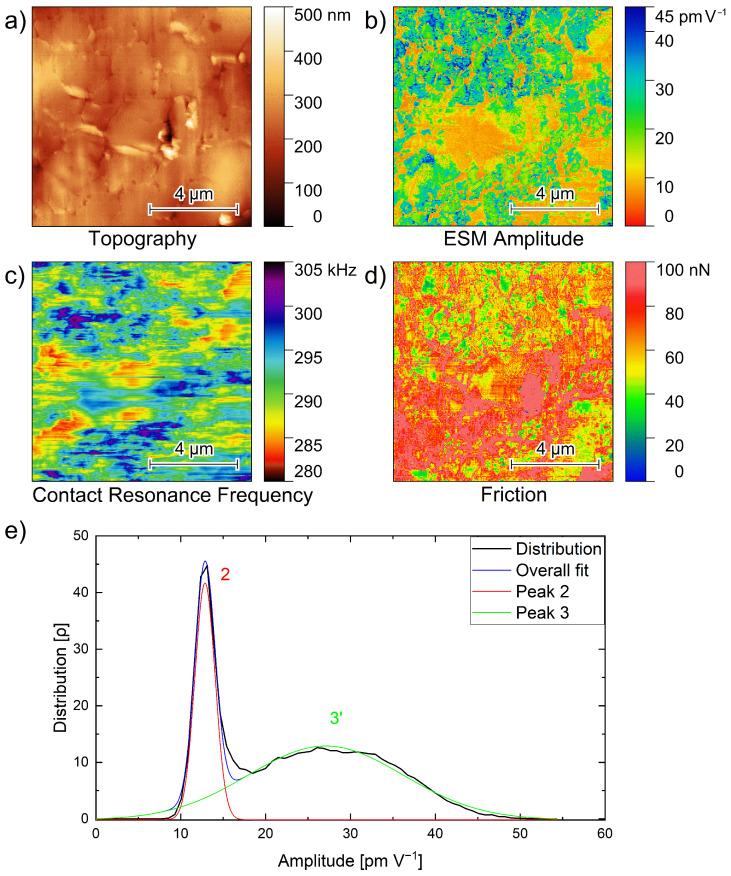
(**a**) Topography, (**b**) amplitude, (**c**) contact resonance frequency, (**d**) friction images and (**e**) amplitude distribution graph of Figure 4b, of a 10 µm × 10 µm area of a PEO_6_–LiTFSI film with 50 wt.% LLZO:Ta film polished with a focused ion beam. Peak 3${}^{\prime }$ reflects the overlapping peaks 3 and 4.

Figure 4 shows the topography, ESM amplitude and ESM frequency images, as well as the amplitude distribution of a 10 µm × 10 µm area on the polished PEO_6_–LiTFSI with 50 wt.% LLZO:Ta film. The topography image in Figure 4a illustrates the surface of the polished area, exhibiting a height difference on the order of 300 nm. A few cracks on the surface, together with vertical lines from the top to the bottom that can be attributed to the curtaining effect, are observed.

The simultaneous recording of the ESM amplitude, the CRF and the friction force signals is very important to gain more information about materials differences than from topographical measurements alone. Therefore, in Figure 4b, the ESM amplitude signal image is shown. At the top part of the image, multiple green and blue spots with higher amplitude values are seen. Additionally, in the bottom part of the image, more green areas are observed.

Orange regions of lower amplitude values are visible in the middle of the image, as well as a few orange regions on the right side. Furthermore, there are areas with lower amplitude values in between and around the green and blue spots. The amplitude distribution of the PEO_6_–LiTFSI electrolyte in Figure 3a shows amplitude values up to 20 pm V^−1^, verifying that the orange areas within the hybrid electrolyte film in Figure 4b reflect the polymer part of the electrolyte.

As the ceramic particles show significantly higher amplitude values, as indicated by the amplitude distribution in Figure 3b, the green and blue spots with amplitude values up to 45 pm V^−1^ are attributed to the ceramic particles. As in Figure 1a, the ceramic particles form agglomerates.

Figure 4c shows the variations of the contact resonance frequency. This is simultaneously recorded with the topography and amplitude image. From the top right and middle to the lower left area decreased CRFs are observed. This is in agreement with the amplitude image in Figure 4b and the friction image in Figure 4d displaying higher friction in the same regions although there is no exact correlation between the individual channels. A possible reason for the weak correlation might be an overlapping thin polymer film that influences the various signals differently.

The graph in Figure 4e illustrates the corresponding amplitude distribution of Figure 4b. Two peaks are found in the amplitude distribution: The narrower peak at 12.8 pm V^−1^ exhibits a FWHM of 2.8 pm V^−1^, while the broader second peak with a maximum at 27.0 pm V^−1^ possesses a FWHM of 21.5 pm V^−1^.

Hence, the first peak is rather similar to the second peak in Figure 3a, i.e., reflecting the higher conductive amorphous polymer phase. This important observation confirms the strategy of incorporating particles into a polymer matrix to prevent crystallization and increase the amorphous fraction of the polymer phase. The second peak 3${}^{\prime }$ of the hybrid sample (Figure 4e) is rather broad and exhibits significantly larger amplitude values, comparable to peaks 3 and 4 in the distribution on the LLZO:Ta pellet in Figure 3b. As this peak is absent in the pure PEO_6_–LiTFSI phase and has a similar position to the peaks on the LLZO:Ta pellet, it is expected to originate from the added ceramic particles.

The obtained ESM amplitudes in the hybrid electrolyte reflect the identical ESM amplitudes as obtained for the individual materials. However, an additional higher amplitude was found corresponding to the interfacial area. This increased lithium ion mobility has not been previously observed by EIS [21] as the interfacial areas are not connected. Thus, the interfacial area around te particles is of particular interest, and we focus on this in the next chapter.

### 3.3. Interfacial Analysis between Ceramic Particles and Bulk Polymer

**Figure 5:**
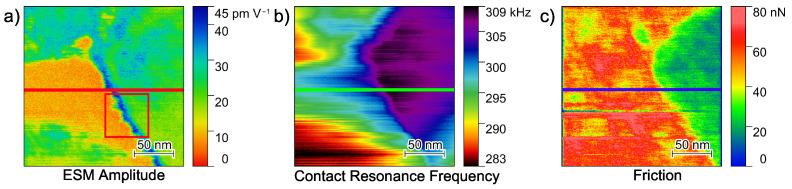
(**a**) Amplitude, (**b**) contact resonance frequency and (**c**) friction force images of a 200 nm × 200 nm area between the bulk PEO_6_–LiTFSI and a single LLZO:Ta particle. The line sections indicated in the middle of the images are shown in Figure 6.

The interfacial area between a single LLZO:Ta particle and the surrounding PEO_6_–LiTFSI is analyzed in greater detail. Figure 5 shows the amplitude signal, Contact Resonance Frequency and friction force images of a 200 nm × 200 nm area of the hybrid electrolyte. With the focus on the interfacial area, Figure 5a displays lower amplitude values on the lower left, while the right hand side exhibits higher amplitude values. The upper left exhibits higher values as well.

The CRF map in Figure 5b illustrates a similar pattern with mostly low CRFs on the left and high CRFs on the right, thus, allowing a clear differentiation between the polymer and ceramic phase. Though subsurface effects on the top left area might influence the amplitude image. The friction force image supports the assignment of the polymer and ceramic phase, with high friction forces on the left and low friction forces on the right-hand side of the map. Therefore, we concluded that the left side of the image shows a bulk polymer phase, while the right side shows a single ceramic particle. In this case, the boundary between both materials is clearly identifiable.

**Figure 6:**
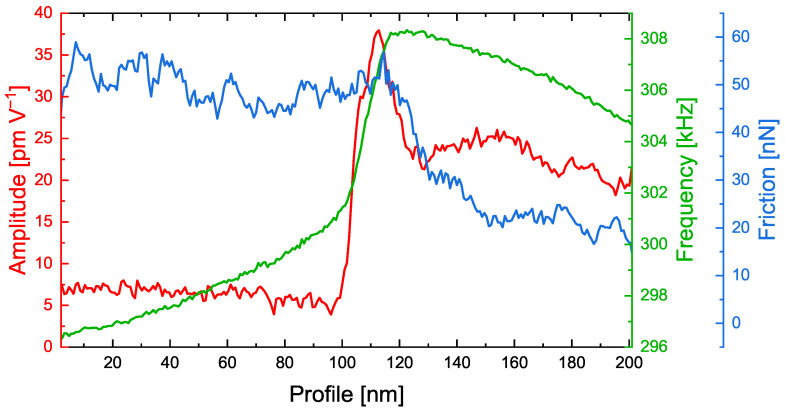
Corresponding line sections as highlighted in Figure 5 of the ESM amplitude, contact resonance frequency and friction force on a PEO_6_–LiTFSI with 50 wt.% LLZO:Ta film. The line sections are averaged over five lines.

It is important to note that the amplitude image shows an area with significantly increased amplitude values at the transition between the bulk polymer phase and the ceramic particle. To assign this region to the polymer or ceramic material, line sections of the amplitude, CRF and friction force at identical positions, are visualized in Figure 6.

As in the images shown in Figure 5, the left side of the graph shows the polymer phase, while the right side shows the ceramic particle. The transition between both materials can easily be recognized between 100 and 120 nm. Interestingly, the amplitude signal exhibits a spike at 110 nm, i.e., exactly in the transition zone. The CRF increases drastically at the same position. However, the friction force decreases at 120 nm and, thus, indicates where the ceramic particle is located.

Combining this information, high friction and a low CRF on the polymer phase and lower friction but higher CRF on the ceramic particle, we verified that the interfacial area with high ESM amplitudes was mostly present on the polymer phase. In agreement with reports from the literature [25,26,31], these findings lead to the major conclusion that, indeed, the transition zone plays an important role in lithium ion transport in PEO_6_–LiTFSI with LLZO:Ta electrolytes. However, the increased ESM amplitude might also reflect the accumulation of lithium ions within this transition zone with a dimension of approximately 20 nm in front of the ceramic particles.

**Figure 7:**
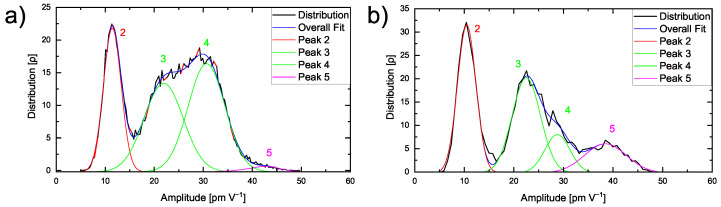
(**a**) Amplitude distribution of 200 nm × 200 nm of a PEO_6_–LiTFSI with 50 wt.% LLZO:Ta film and (**b**) of a smaller region as indicated by the red square shown in Figure 5a.

The effect of the transition zone is also reflected in the amplitude distribution of the region from Figure 5a as shown in Figure 7.

The distribution in Figure 7a displays four distinguishable peaks. While the first peak 2 appears at around 11.6 pm V^−1^ and reflects the amorphous polymer phase as discussed above, the second peak 3 at 22.0 pm V^−1^ and the third peak 4 at 30.7 pm V^−1^ correspond to the ceramic particle, comparable to Figure 3b.

In comparison to the single peak observed in Figure 4e, the high spatial resolution reveals even small variations in the ESM amplitude signal within the hybrid electrolyte. The rather small fourth peak 5 at 42.9 pm V^−1^ is only obtained at sufficiently high spatial resolution and is attributed to the interfacial area exhibiting very high ESM amplitudes. This is verified by focusing on the interfacial area and highlighted in Figure 7b, where the amplitude distribution according to the highlighted area in Figure 5a is illustrated.

A significantly enlarged peak 5 is found. The obtained amplitude distributions indicate that, in Figure 4e, a fourth peak might be hidden in the amplitude values distributed around 40 to 50 pm V^−1^. However, on larger scale images, the resolution is not sufficient to visualize the transition zone of 20 nm. The increased amplitude signal validates the higher lithium mobility inside the interfacial area, while a transport process between an amorphous polymer and ceramic is possible.

These results underline the importance of the interaction between ceramic particles and the polymer matrix to increase the overall conductivity. The outstanding influence of interfacial transition zones, reminiscent of the space charge areas between the polymer phase and embedded ceramic particles, as discussed in the literature [29,30,31], are confirmed.

## 4. Conclusions

A PEO_6_–LiTFSI with 50 wt.% LLZO:Ta all-solid-state hybrid electrolyte film was examined by means of Electrochemical Strain Microscopy. We demonstrated that the individual materials exhibited significantly different signals in the friction force, Contact Resonance Frequency and amplitude signals. The polymer electrolyte exhibited a higher friction force with lower CRFs as expected. In agreement with the literature, higher ionic conductivity inside the ceramic led to significantly higher ESM amplitude values compared to the bulk polymer phase.

The amplitude distribution of a pure PEO_6_–LiTFSI polymer electrolyte exhibited two peaks that can be attributed to crystalline (peak 1) and amorphous (peak 2) regions. The distribution on a LLZO:Ta pellet showed two peaks at higher amplitude values. Within the hybrid electrolyte film, those peaks were similar except for peak 1, as the introduction of particles suppresses the crystallization. High-resolution imaging revealed a region around the ceramic particles with increased amplitude values even higher than those of bulk LLZO:Ta, thereby, indicating the accumulation of lithium ions.

**Figure 8:**
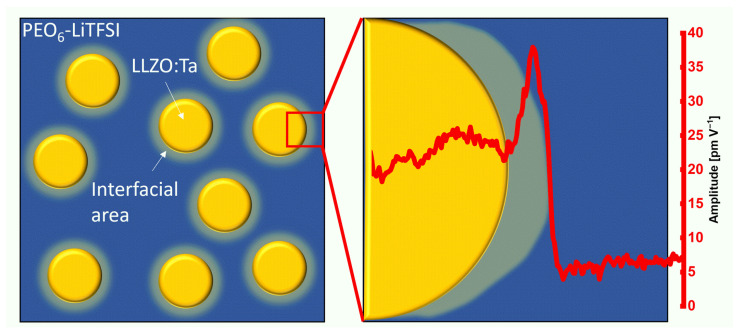
Schematic illustration of the bulk polymer phase with several ceramic particles and a segment of a single particle with the ESM amplitude line section of Figure 6. The interfacial area shown is not to scale.

This interfacial area has a dimension of 20 nm inside the polymer phase adjacent to the ceramic particle as verified by friction force and CRF and schematically depicted in Figure 8. In the distribution of the amplitude values, another peak was observed that is attributed to this interfacial area.

The results presented in this manuscript are of significant importance to obtain a further understanding of the ionic transport mechanisms inside hybrid electrolytes. Based on these results, we propose two strategies for the further improvement of hybrid solid-state electrolytes.

First, coating the particles to decrease the interfacial resistance might open a path to take advantage of the intrinsically higher ionic conductivity of the ceramic. Second, to capitalize on the high lithium content of the interfacial area and increase the overall ionic conductivity of the hybrid electrolyte, a system with a continuous percolation path along the ceramic particles should be created. Both aspects are subject to further investigations.

## Data Availability

The data presented in this study are available on request from the corresponding author.

## Abbreviations

The following abbreviations are used in this manuscript:

ESM	Electrochemical Strain Microscopy
AFM	Atomic Force Microscopy
ASSB	All-Solid-State Battery
FIB	Focused Ion Beam
SEM	Scanning Electron Microscopy
CRF	Contact Resonance Frequency
NMR	Nuclear Magnetic Resonance
BSE	Back Scattered Electron
